# Fuzzy clustering and distributed model for streamflow estimation in ungauged watersheds

**DOI:** 10.1038/s41598-021-87691-0

**Published:** 2021-04-15

**Authors:** Amirhosein Mosavi, Mohammad Golshan, Bahram Choubin, Alan D. Ziegler, Shahram Khalighi Sigaroodi, Fan Zhang, Adrienn A. Dineva

**Affiliations:** 1 Environmental Quality, Atmospheric Science and Climate Change Research Group, Ton Duc Thang University, Ho Chi Minh City, Vietnam; 2 Faculty of Environment and Labour Safety, Ton Duc Thang University, Ho Chi Minh City, Vietnam; 3 Watershed Management Department, Natural Resources and Watershed Management Office, Astara, Iran; 4 Soil Conservation and Watershed Management Research Department, West Azarbaijan Agricultural and Natural Resources Research and Education Center, AREEO, Urmia, Iran; 5 Faculty of Fisheries and Aquatic Resources, Mae Jo University, Chiang Mai, Thailand; 6 Reclamation of Arid and Mountainous Regions Department, Faculty of Natural Resources, University of Tehran, Karaj, Iran; 7 Key Laboratory of Tibetan Environment Changes and Land Surface Processes, Institute of Tibetan Plateau Research, Chinese Academy of Sciences, P.O. Box 2871, Beijing, 100085 China; 8 Institute of Research and Development, Duy Tan University, Da Nang, 550000 Vietnam; 9 John von Neumann Faculty of Informatics, Obuda University, 1034 Budapest, Hungary

**Keywords:** Environmental sciences, Hydrology

## Abstract

This paper proposes a regionalization method for streamflow prediction in ungauged watersheds in the 7461 km^2^ area above the Gharehsoo Hydrometry Station in the Ardabil Province, in the north of Iran. First, the Fuzzy c-means clustering method (FCM) was used to divide 46 gauged (19) and ungauged (27) watersheds into homogenous groups based on a variety of topographical and climatic factors. After identifying the homogenous watersheds, the Soil and Water Assessment Tool (SWAT) was calibrated and validated using data from the gauged watersheds in each group. The calibrated parameters were then tested in another gauged watershed that we considered as a pseudo ungauged watershed in each group. Values of R-Squared and Nash–Sutcliffe efficiency (NSE) were both ≥ 0.70 during the calibration and validation phases; and ≥ 0.80 and ≥ 0.74, respectively, during the testing in the pseudo ungauged watersheds. Based on these metrics, the validated regional models demonstrated a satisfactory result for predicting streamflow in the ungauged watersheds within each group. These models are important for managing stream quantity and quality in the intensive agriculture study area.

## Introduction

Ungauged watersheds are prevalent throughout the world^1^, particularly in small watersheds where a strong understanding of local stream response to rainfall inputs is critical to specific management issues^2^. While a prediction of streamflow and value of output in a specific time is essential for engineering practices and having sustainable watershed management. Also, streamflow value in a basin output is a reliable diagnostic variable showing the impact of factors such as climate changes, land-use changes, and human activities that act on a given basin. The International Association of Hydrological Sciences (IAHS) decade on prediction/forecasting in the unmonitored basin is an initiative^3^ focused on predicting streamflow through a variety of techniques such as radar altimetry^4–6^, regression methods^7–9^, and physical similarity approaches^10,11^. In general, assessment of streamflow in ungauged watersheds is a challenging task^12,13^ because of the difficulty of obtaining reliable data from similar gauged watersheds of the appropriate spatial and temporal scales, as well as representative watershed properties, to develop reliable prediction models^3,14^.

Over the years, hydrology engineers have developed numerous hydrological models that allow having information about the water cycle in a basin. Hydrological modeling of the water cycle in basins with speed response to rainfall such as small watersheds or distributed watersheds is necessary for water resource management^15^. The most widely used tools, such as the Soil and Water Assessment Tool (SWAT) has been successfully implemented for simulating streamflow under varying land use and climate conditions in numerous watersheds throughout the world^16–20^. The SWAT model has numerous parameters that can be reduced for regionalization objectives, meanwhile preserving prediction skills^14,21,22^.

The area occupying the center of the Ardabil Province of Iran is a high-yield agriculture production region where recent human activities and land degradation have contributed to accelerated erosion and increased flood frequency^23^. These factors have reduced fertility, lowered the volume of available water, and degraded the water resource reservoirs. All these impacts threaten the agricultural sustainability of region^24^. Addressing these issues requires an assessment of relevant runoff, erosion, and sediment transport processes to guide water resource planning, hazard assessments, and catchment management. Hydrological models are useful management tools in watersheds where streamflow and sediment yields are tied to natural phenomena, anthropogenic activity, and climate variability^13,25^. However, models require data for calibration and testing—and lack of data is the main limitation for applying them in regions such as Ardabil Province where many watersheds are ungauged, having no hydrological monitoring stations.

Therefore, the main objective of the work is to develop reliable predictions that can guide management in the ungauged watersheds of this important agriculture region in Iran. This study develops a regionalization approach based on the SWAT model for estimating streamflow in the ungauged watersheds in the Ardabil Province, Iran. To achieve this objective, we applied the FCM method to group 46 watersheds based on their similarity in a variety of properties (such as area, maximum elevation, minimum elevation, slope length, slope, rainfall, and temperature); and used the SWAT model for streamflow regionalization objectives in each homogenous region.

## Materials and methods

### Study area

**Figure 1:**
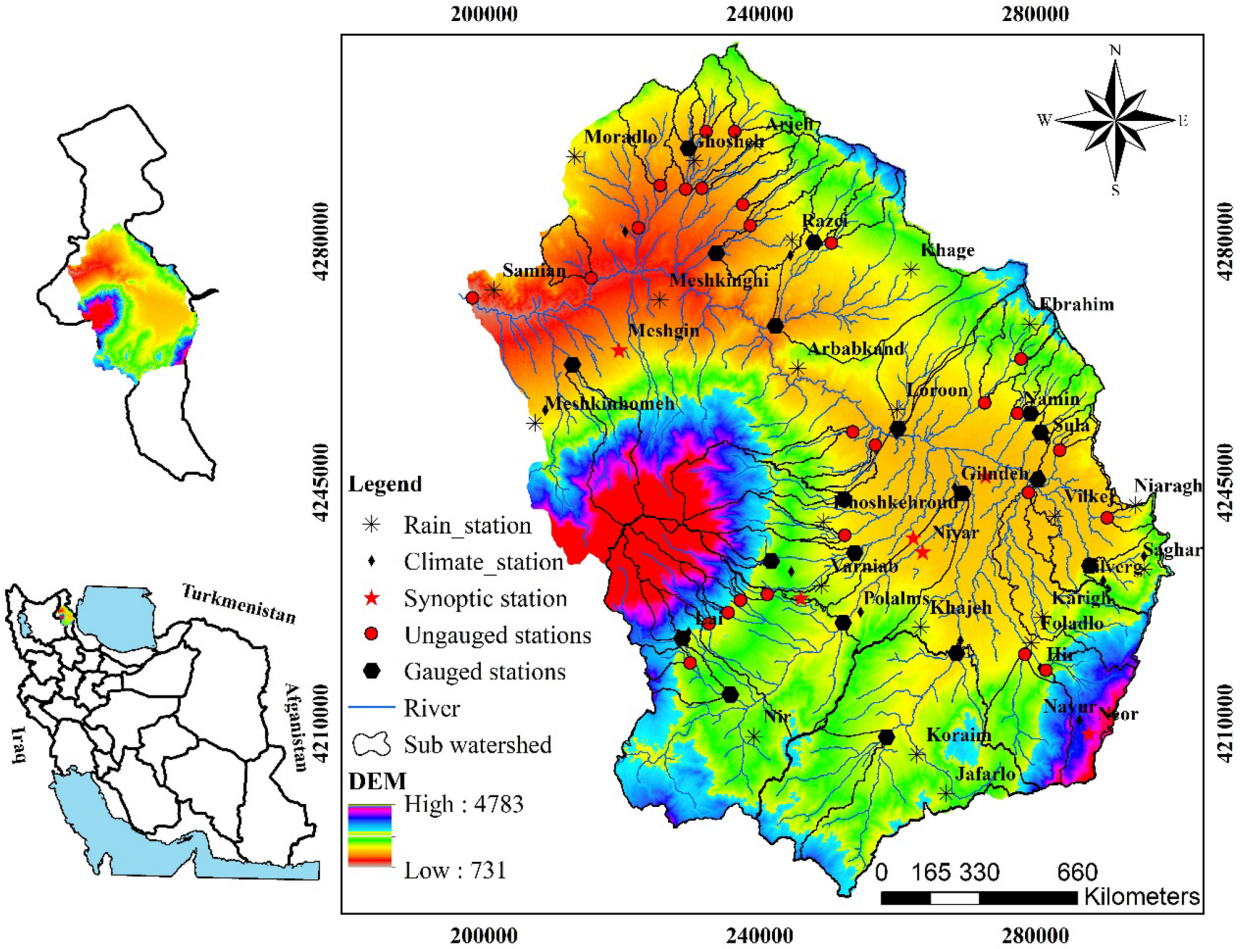
Delineated watersheds for gauged and ungauged stations in the Gharehsoo area. Black Hexagonal shows the gauged station, red circles show the ungauged station, red star marks show synoptic stations, black stars show rain stations, black diamonds show climate station and blue lines show river. The map was generated using ArcGIS Desktop 10.3, https://desktop.arcgis.com/en.

The area of interest is the headwaters of the Gharehsoo River in the Ardabil Province, Iran (Fig. 1). The 287-km river flows past many cities before entering the Aras River, then the Caspian Sea. The river system provides water for domestic, industrial, agriculture, fishing sectors that have about 1.25 million users^23^. In upstream, the river is formed from outflows from basins in the Talesh, Bozgosh, and Sabalan mountains.

The highest elevation in the study area is 4783 m a.s.l. in the Sabaln Mountain; the lowest elevation is 731 m a.s.l. in the outlet of the watershed. The area is dominated by a semi-arid climate with a mean annual rainfall of 340 mm, although there is uncertainty associated with precipitation totals at high elevations. May and August are the wettest (41 mm) and the driest (6 mm) months of the region, respectively^26^. This area is one of the coldest areas in Iran, having an average annual temperature near 9 °C and 130 days of freezing in a year.

The geology is dominated by the Quaternary rocks and sediments, and the soils are typically Inceptisols. The central parts of the research area with low slope and deep soil are important because of agriculture products. Potato and wheat are the main products in the studded area where about 50% of people live in rural areas and work on farms^23^. In the study area, the rangeland is mainly covered by perennial grasses (27%) and perennial forbs (13%), the two dominant vegetation types in semi-steppe lands^27^.

### SWAT model

We used the physics-based and semi-distributed Soil and Water Assessment Tool (SWAT), developed by the USDA Agricultural Research Service^28^. SWAT uses hydro-meteorological and topographic information to simulate streamflow and is therefore useful for management purposes related to runoff calculations, sediment load estimation, and agricultural production^29–31^. Simulation of catchment hydrology in SWAT considers the land and river routing phases of the hydrology cycle. The first part controls the amount of water transported to the channel in each sub-watershed. The second relates to the flow through the channel network to the catchment outlet^32^. The SWAT model divides a catchment into sub-basins based on a DEM (digital elevation map). Maps are added regarding land use, topography, and soil, which allow further delineation and creation of hydrological response units (HRU_s_) to represent a catchment^15,33^.

The SWAT model is composed of various mathematical equations and empirical formulas that are used for determining parameters at daily, monthly, or annual time steps^34^. For example, we employ the Soil Conservation Service (SCS) Curve Number^35^ equation for estimating surface runoff:

1 $$ Q_{{{\text{surf}}}} = \frac{{(R_{{{\text{day}}}} { - 0}{\text{.2 S}})^{2} }}{{\left( {R_{{{\text{day}}}} + 0.8S} \right)}} $$

where ${\text{Q}}_{{{\text{surf}}}}$ is surface runoff elevation (mm); ${\text{R}}_{{{\text{day}}}}$ is daily rainfall (mm); *S* is the soil surface moisture (mm). Runoff occurs when *R*_*day*_ is greater than 0.5^36^. The *S* parameter varies based on the soil type, slope of the catchment, and land use management^35^:

2 $$ S = 25.4\left( {\frac{1000}{{CN}} - 10} \right) $$

where, *CN* is a dimensionless parameter ranging from 0 to 100, which is determined by the SWAT from the input parameters.

The Manning coefficient is used to determine the rate and velocity of streamflow. Finally, we used the Muskingum River routing method to handle variable storage and river routing of water.

To calculate potential evapotranspiration, we used the Penman–Monteith Equation^32^:

3 $$ \lambda E = \frac{{\Delta \cdot \left( {H_{net} - G} \right) + \rho_{air} \cdot c_{p} \cdot \left[ {e_{z}^{o} } \right]/r_{a} }}{{\Delta + \gamma \cdot \left( {1 + r_{c} /r_{a} } \right)}} $$

where $\lambda E$ is the latent heat flux density (MJ m^−2^ day^−1^); *E* is the depth rate of evaporation (mm day^−1^); ∆ shows the saturation vapor pressure–temperature curve (kPa °C^−1^); $H_{net}$ is the net radiation (MJ M^−2^ day^−1^); $G$ is the heat flux density to the ground (MJ M^−2^ day^−1^); $e_{z}^{o}$ is the saturation vapor pressure of air at height ${\text{z}}$ (kPa); $\gamma$ is the psychometric constant (kPa °C^−1^); $r_{c}$ is the plant canopy resistance (s m^−1^); $r_{a}$ is the diffusion resistance of the air layer (aerodynamic resistance) (s m^−1^); $\rho_{air}$ is dry air density; and $C_{p}$ is specific heat capacity of air (J kg^−1^ K^−1^).

### Watershed characteristics

Before implementing the SWAT model, we selected seven parameters for 19 gauged and 27 ungauged watersheds according to the literature review and data availability: area, maximum elevation, minimum elevation, slope length, slope, rainfall, and temperature. These parameters have been widely used for watershed clustering in hydrological studies^7,37–39^. In each watershed, the maximum elevation, minimum elevation, and slope parameters were calculated using an ASTER DEM map (with 30 × 30 m resolution), and the average in space for rainfall and temperature (from 1985 to 2015) were calculated using the weights calculated by the Voronoi maps^12^ in the ArcGIS 10.5. The number of parameters is high compared with other regionalization applications^7,37–39^. The area of the watersheds ranges between 8 (SW 39) and 1194 km^2^ (SW 33). The ranges of rainfall and temperature are considerable. In the time period of 1985–2015, the minimum average rainfall (264 mm) is found in SW27 in the east of the region; and the maximum average rainfall (487 mm) occurs in SW19 in the west. In this period, the temperature range is between 7.3 and 13.0 °C, indicating a cold-weather regime. The mountainous topography contributes to a high elevation range: 731–4783 m. The minimum average catchment slope is 4.2% in SW9; and the average maximum slope is 34.5% in SW19. Slope length in SW33 (161 km) is much higher than in other watersheds: SW27 has the smallest slope length (6.6 km). All of these data were used for watershed grouping via clustering and individual watershed simulations.

### Fuzzy c-means clustering

Watersheds with the same climate and topographic circumstances often have the same hydrological conditions, and therefore, they can be grouped by clustering, thereby providing a means of developing validated models for ungauged river systems^13,40^. Indeed, clustering is a pre-processing step for regionalization objectives. Clustering methods divide a data set into classes that similar objects are in an equal group. Among the clustering methods, soft clustering methods such as the Fuzzy c-means (FCM) technique reduces the uncertainty in identifying the members of the group by assigning a degree of membership for each member in each group^41,42^. Therefore, we used the FCM method for identifying similar donor basins to the ungauged basins. In this method, the degrees of membership for each watershed in each group is determined using fuzzy memberships^43^. This algorithm has been widely used for clustering similar watersheds for several applications^41,42^. The algorithm, created by Dunn^44^, and improved with Bezdek^45^, is represented by the following objective function:

4 $$ J_{m} = \mathop \sum \limits_{i = 1}^{N} \mathop \sum \limits_{j = 1}^{C} U_{ij}^{m} x_{i} - v_{j}^{2} \quad {1} \le {\text{ m }} < \, \infty $$

with the restraint in the form

5 $$ \mathop \sum \limits_{i = 1}^{c} u_{ij} = 1\quad {\text{for}}\,\,j = 1, 2, \ldots , n $$

where *m* is a fuzzification parameter (number greater than 1); *n* is the number of data points; *C* is the cluster number considered; *U*_*ij*_ is the membership of pixel *x*_*i*_ in the *i*th cluster, and *v*_*j*_ is the *j*th cluster number.

As some parameters require standardizing before clustering to eliminate a heterogeneous effect of different units^42^, we normalized them to a range of 0 to 1 as follows:

6 $$ X_{norm} = \frac{{x - x_{min} }}{{x_{max} - x_{min} }} $$

where $X_{norm}$ is the normalized value, $x$ is the initial value, $x_{min}$ and $x_{max}$ are the minimum and maximum values.

As the number of clusters is not pre-defined, the optimum clustering number was selected through an iterative process that is repeated until the optimization criteria are met. Our criteria were maximization of the partition coefficient ($V_{PC}$) and minimization of the partition entropy ($V_{PE}$), which are calculated as follows^45^:

7 $$ V_{PC} = \frac{1}{n}\left( {\mathop \sum \limits_{j = 1}^{n} \mathop \sum \limits_{i = 1}^{c} = u_{ij}^{2} } \right) $$

8 $$ V_{PE} = - \frac{1}{n}\left( {\mathop \sum \limits_{j = 1}^{n} \mathop \sum \limits_{i = 1}^{c} u_{ij} \log (u_{ij} )} \right) $$

where *n* and *c* are number of data points and clusters, respectively, $u_{ij}$ is the degree of membership of *x*_*i*_ in the cluster *j*. The ranges of $V_{PC}$ and $V_{PE}$ are between 1/*c* to 1 and 0 to $log_{2}^{c}$, respectively.

The processes of the FCM can be explained in: standardizing the data set, choosing m and c, calculate partition matrix ($u_{ij}$), and detecting the number of clusters for a successful clustering.

### SWAT forcing data

Hydro-meteorological forcing data for the SWAT model consists of rainfall, minimum and maximum temperature, relative humidity, wind speed, and solar radiation with daily time step that covered 30 years period from January 1985 to December 2015. Rainfall data were derived from 27 stations maintained by the Iran Water Resources Management Company (IWRMC), and eight climate stations managed by the Iran Meteorological Organization (IMO). Wind speed, minimum and maximum temperature, relative humidity, and solar radiation were associated with the IMO climatic and synoptic stations. Missing values were filled using linear regression. The corrected data were prepared in the ArcSWAT2012 format and defined as model inputs. Daily stream discharge values (1985–2015) for model calibration/testing were associated with only six IWRMC hydrometric stations.

The spatially distributed layers for input into the SWAT include a DEM, a soil map, and a land-use map. The DEM is the raster layer consisting of a range of elevation values for each pixel. We used a DEM with a 30 × 30 m resolution, which was downloaded from the ASTER Global Digital Elevation Model (ASTGTM) in four sheets (N38E47, N37E47, N38E48, and N37E48) and merged. The map projection was Universal Transverse Mercator (UTM) on the spheroid of WGS84; it was processed in the first step of the SWAT execution. Sub-watershed characteristics such as slope gradient, slope length, and details of the stream network were determined from the DEM.

We made the land-use map using the ENVI 5.2 software and Landsat 8 satellite imagery available at the US Geographical Survey (www.usgs.gov). The real ground truth points were identified randomly from the high accuracy of the map. According to the literature, the land-use changes are not considerable in the model calibration and validation periods (i.e., 2004–2014)^27,34^. The soil layer was obtained from the Ardabil Department of Natural Resource and completed with the FAO digital soil map of Iran^46^. Information about the soil’s polygons such as the number of layers, hydrological soil groups, soil texture was imported to the SWAT database in Access format. The soil characteristics of the study area are presented in Table 1.

**Table 1:** Soil characteristics in the case study.

Type	NL	HSG	Depth (mm)	SHC (mm/h)	Soil texture	OCC (%)	AWC (mm/mm)	MBD (Mg/m^3^)
1	3	A	1000	324	Sand	0.1	0.125	1.6
2	2	B	870	108	Loamy sand	4.4	0.172	0.8
3	2	C	690	16.69	Loam	0.7	0.122	1.2
4	2	D	780	2.68	Loam	0.7	0.097	1.5

*NL* number of layers, *HSG* hydrological soil group, *SHC* saturated hydraulic conductivity, *OCC* organic carbon content, *AWC* available water capacity, *MBD* moist bulk density.

### Calibration and validation of the SWAT model

In each cluster, two gauged watersheds that have a high degree of membership and sufficient data were used for simulation with the SWAT model. One of these gauged stations is considered as the “donor” watershed, and another is the ‘pseudo’ ungauged watershed. The donor watershed is used to parameterize and calibrate the SWAT model parameters. The pseudo ungauged watershed is used to test the calibrated model. More details/descriptions of the donor and pseudo ungauged watersheds can be found in Choubin et al.^12^. For assessing the model performance, the determination coefficient (R^2^), Nash–Sutcliffe efficiency (NSE), root mean square error (RMSE), and relative BIAS (RBIAS) indices were used. The related equations for these indexes are in Mahmood et al. (2017).

**Figure 2:**
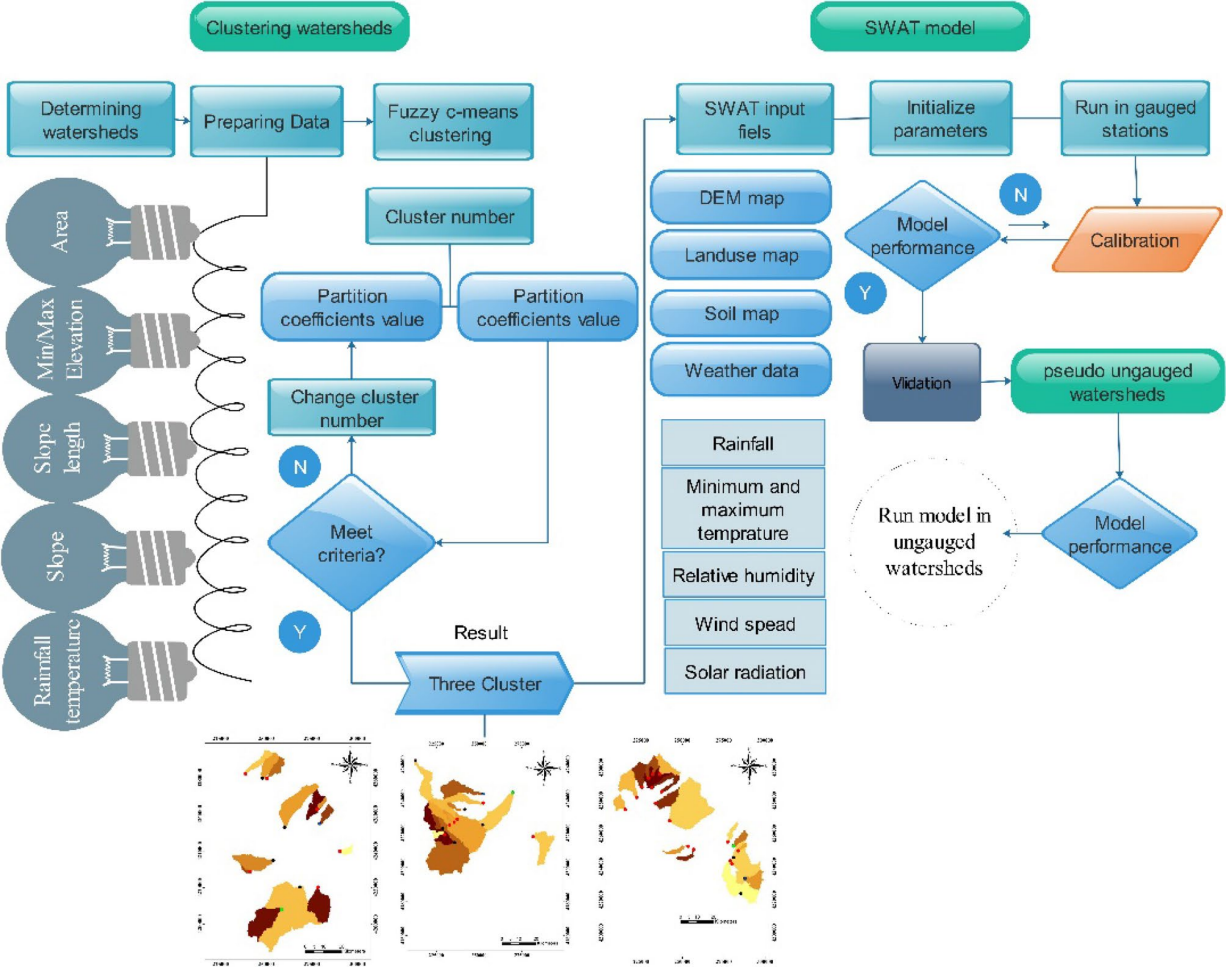
A schematic framework of the research steps. The maps were generated using ArcGIS Desktop 10.3, https://desktop.arcgis.com/en.

In the SWAT Calibration Uncertainty Procedure (SWAT-CUP) software, we used a sequential uncertainty fitting (SUFI-2) algorithm for sensitivity analysis and model calibration and validation. This algorithm is an inverse semi-automatic reversed model that has high performance when calibrating many parameters^34,47^. For sensitivity analysis of the parameters, we used SWAT-CUP, which is based on the SUFI-2 algorithm. For each donor watershed, the periods 2004–2011 and 2012–2014 were used for calibration and validation, respectively. During the calibration, we determined optimal values for all sensitive parameters. In the validation period, the model was executed for watersheds using the optimal parameter value. In testing the model on the pseudo ungauged watersheds, the model was run with calibrated parameters for the period 2004–2011. A flowchart of research steps is shown in Fig. 2.

## Results and discussion

### Homogeneous regions

**Figure 3:**
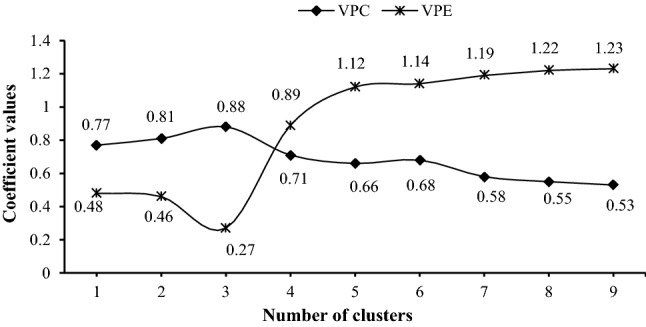
The VPC and VPE coefficient values in each cluster for detecting best number of classes by the FCM method.

**Figure 4:**
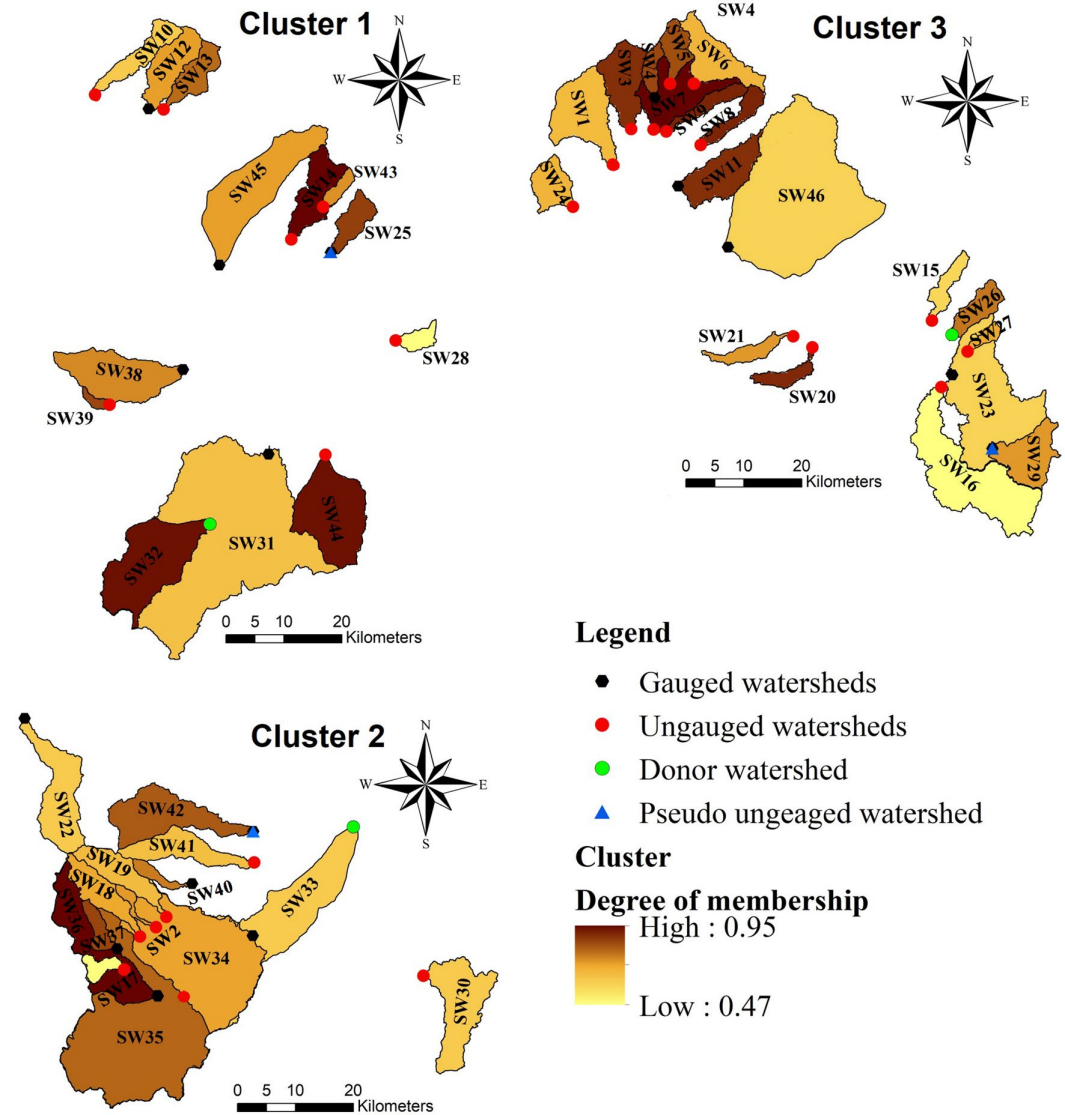
Gauged, ungauged, donor, and pseudo ungauged watersheds, and degree of membership in each cluster. The map was generated using ArcGIS Desktop 10.3, https://desktop.arcgis.com/en.

The maximized partition coefficients (V_PC_) and minimized partition entropy values (V_PE_) indicated three homogeneous clustering groups with 13, 14, and 19 members (Table 2; Fig. 3). Degree of membership in each cluster ranged from 0.46 to 0.82, 0.47 to 0.96, and 0.48 to 0.95, respectively. The first cluster includes watersheds with low elevation and mostly plain areas in the western part of Gharehsoo Watershed (Fig. 4). Watersheds in the second cluster are located in the southeast part with high elevation mountains. Watersheds in the third cluster are located in the south and east parts of the region with intermediate elevation (Fig. 4). Mean monthly temperature and mean annual rainfall from 1985 to 2015 time period are listed in Table 3. In these three clusters are 13, 14, and 18 watersheds, of which 7, 6, and 13 watersheds are ungauged, respectively.

**Table 2:** Number of clusters and degree of membership (DM) for each watershed.

Watershed	Cluster	DM	Watershed	Cluster	DM	Watershed	Cluster	DM
SW10^a^	1	0.58	SW2^a^	2	0.75	SW1^a^	3	0.86
SW12	1	0.76	SW17^a^	2	0.47	SW3^a^	3	0.89
SW13^a^	1	0.79	SW18^a^	2	0.73	SW4	3	0.85
SW14^a^	1	0.82	SW19^a^	2	0.73	SW5^a^	3	0.84
SW25	1	0.65	SW22	2	0.69	SW6^a^	3	0.67
SW28^a^	1	0.48	SW30^a^	2	0.52	SW7^a^	3	0.95
SW31	1	0.60	SW33	2	0.54	SW8^a^	3	0.91
SW32	1	0.81	SW34	2	0.64	SW9^a^	3	0.91
SW38	1	0.46	SW35^a^	2	0.74	SW11	3	0.89
SW39^a^	1	0.65	SW36	2	0.82	SW15^a^	3	0.58
SW43^a^	1	0.67	SW37	2	0.95	SW16^a^	3	0.48
SW44^a^	1	0.81	SW40	2	0.47	SW20^a^	3	0.90
SW45	1	0.66	SW41^a^	2	0.79	SW21^a^	3	0.73
			SW42	2	0.67	SW23	3	0.59
–	–	–	–	–	–	SW24^a^	3	0.77
–	–	–	–	–	–	SW26	3	0.48
–	–	–	–	–	–	SW27^a^	3	0.51
						SW29	3	0.73
–	–	–	–	–	–	SW46^a^	3	0.49

^a^Ungauged watershed.

**Table 3:** Statistical summary of the monthly average air temperature (from 1985 to 2015) in three modeled donors’ watersheds and mean weighted rainfall from 32 stations in the Gharehsoo Watershed.

Month	Temperature (°C)			Weighted rainfall (mm)
	Cluster 1 (SW32)	Cluster 2 (SW33)	Cluster 3 (SW26)	Gharehsoo watershed
Jan	− 2.98	2.53	− 0.20	20.91
Feb	− 3.54	2.16	− 0.81	23.31
Mar	− 0.67	4.78	2.59	27.98
Apr	4.96	9.27	7.90	39.05
May	8.81	13.69	12.09	56.73
Jun	14.42	18.83	15.69	30.53
Jul	15.73	21.54	18.21	10.97
Aug	16.68	23.17	18.97	6.64
Sep	14.79	19.84	16.45	14.24
Oct	10.85	15.57	13.26	23.93
Nov	4.80	9.39	8.21	36.57
Dec	− 0.45	2.53	2.84	24.98
Average	6.95	12.1	9.6	26.32
Maximum	16.68	23.17	18.97	56.73
Minimum	− 3.54	2.16	0.81	6.64
SD	7.61	7.68	7.19	14.12

Based on the FCM results (Table 2), the SW32 was selected as the donor watershed in the first cluster; and SW25 as the pseudo ungauged watershed (Fig. 4). In the second cluster, the SW33 was selected as the donor watershed and the SW42 as the pseudo ungauged watershed. Finally, in the third cluster, the SW26 was selected as the donor watershed and the SW29 as the pseudo ungauged watershed (Fig. 4). It should be noted that the selected donor/pseudo ungauged watersheds have sufficient climatic data rather than others (for streamflow simulation by the SWAT model).

### Sensitivity analysis results

The global sensitivity analysis performed using the SUFI2 algorithm in each donor watershed. Parameters that have p-value < 0.05 and a high t-stat value (modulus) were considered sensitive. In each cluster, we identified six sensitive parameters for simulating streamflow (Table 4). In all clusters, the curve number (CN) parameter had the highest sensitivity, a result that is consistent with other studies in Iran^34,48^. In the first cluster, with smooth topography, groundwater parameters (GW-Delaye, GE-Revap, Rchrg_DP) and soil parameters (Sol_Awc, Sol_Z) were the most sensitive^15^. In the second cluster, which includes mountains, the Sol-K is one of the sensitive parameters^13^. In the third cluster, the Esco parameter is the second sensitive parameter that shows in this cluster the evapotranspiration has a considerable effect on runoff.

**Table 4:** Results of the optimized parameters for each cluster.

Cluster	Parameter name	Max value	Min value	Fitted value	P-value	t-stat
1	Soil run-off curve number for moister condition II (CN)	90	40	72	0.00	− 6.86
	Groundwater delay time (Gw_Delay)	150	1	28	0.00	− 4.28
	Groundwater revamp. Coefficient (GE-Revap)	0.9	0.01	0.095	0.00	− 2.51
	Soil available water storage capacity (Sol_Awc)	1	0	0.38	0.02	1.73
	Deep aquifer percolation fraction (Rchrg_DP)	1	0.01	0.01	0.02	− 1.57
	Depth from soil surface to bottom of layer (Sol_Z)	500	50	80	0.05	− 1.56
2	Soil run-off curve number for moister condition II (CN)	90	40	65	0.00	12.35
	Soil conductivity (Sol-K)	80	4	21	0.02	3.24
	Groundwater revamp. Coefficient (GE-Revap)	0.9	0.01	0.21	0.03	− 2.57
	Soil available water storage capacity (Sol_Awc)	1	0	0.24	0.03	2.63
	Plant uptake compensation factor (Epco)	1	0	0.41	0.03	− 2.11
	Snow melt base temperature (SMTMP)	5	0	0.187	0.05	− 2.02
3	Soil run-off curve number for moister condition II (CN)	90	40	78	0.00	− 5.63
	Soil evaporation compensation factor (Esco)	1	0	0.32	0.00	− 3.99
	Base flow alpha factor (Alpha_bf)	1	0	0.39	0.03	1.62
	Groundwater revamp. Coefficient (GE-Revap)	1	0.01	0.09	0.04	− 1.58
	Deep aquifer percolation fraction (Rchrg_DP)	1	0.01	0.63	0.05	− 1.58
	Temperature laps rate (Tlaps)	20	0	4.75	0.05	− 1.50

### Model calibration and testing results

**Figure 5:**
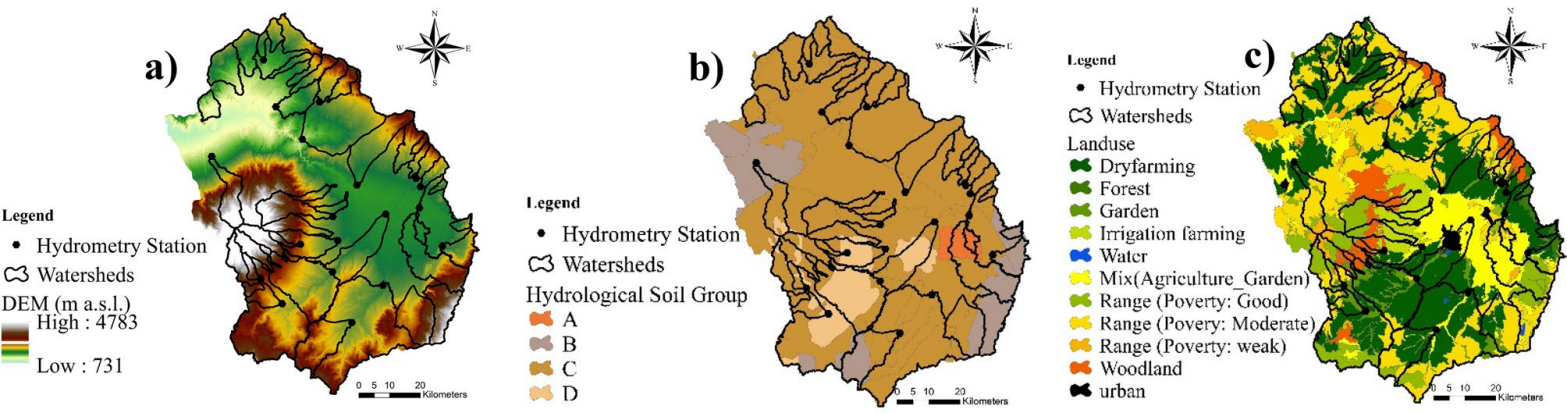
General map of the case study: (**a**) DEM, (**b**) hydrological soil group, (**c**) land use. The maps were generated using ArcGIS Desktop 10.3, https://desktop.arcgis.com/en.

**Figure 6:**
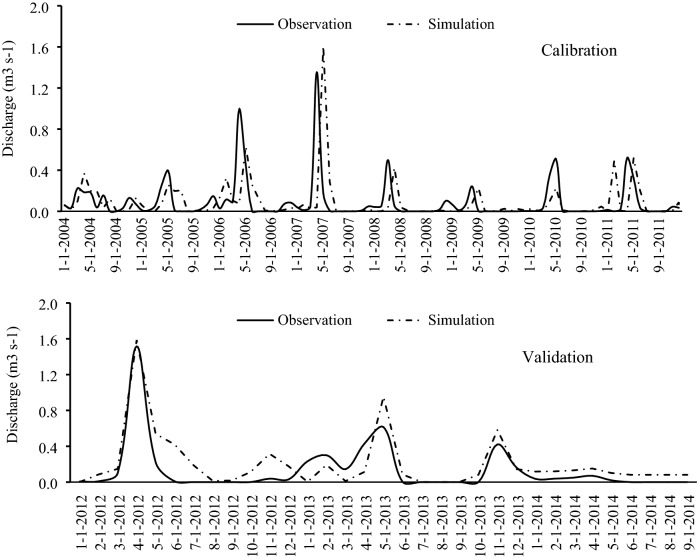
Streamflow simulation in the first cluster in SW32 station.

**Figure 7:**
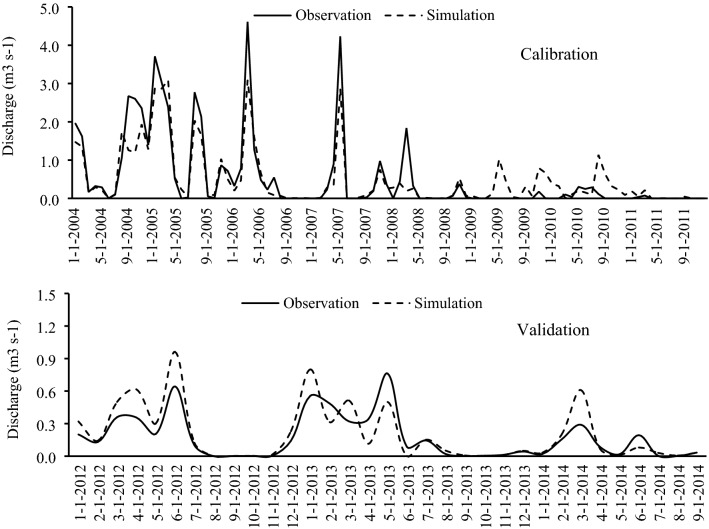
Streamflow simulation in the second group in SW33 station.

**Figure 8:**
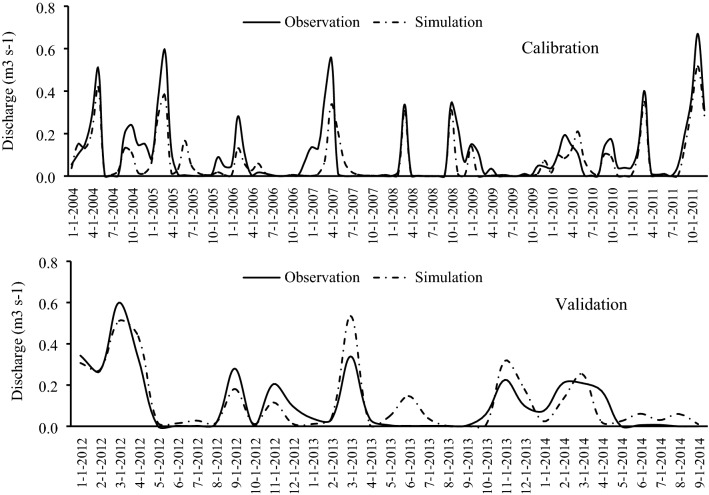
Streamflow simulation in the third group in SW26 station.

**Figure 9:**
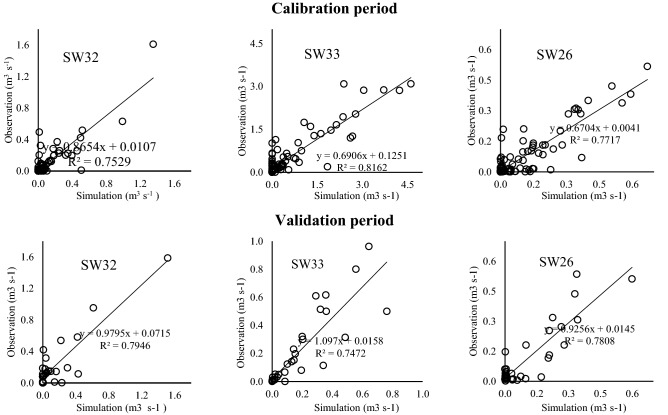
Calculated correlation coefficient in the donor watersheds.

The maps (Fig. 5) and data were imported to the SWAT model and the model was calibrated and validated on three donor watersheds (SW32, SW33, and SW26) for the period 2004–2011 (Figs. 6, 7, and 8). Based on prior studies, we considered results to be acceptable if R^2^ > 0.6^36,49–52^ and NSE > 0.6^25,53,54^. The R^2^ and NSE values were both ≥ 0.7 during the calibration phase, indicating an optimal set of parameters had been achieved (Table 5; Fig. 9). The values of the RMSE are high compared with observations, this is mostly because of bad prediction in low flows.

**Table 5:** Evaluation metrics in the donor and pseudo ungauged watersheds.

Watershed type	Period	Stations	Metrics			
			R^2^	NSE	RMSE	RBIAS
Donor watershed	Calibration	Group 1 (SW32)	0.75	0.73	1.01	0.014
		Group 2 (SW33)	0.82	0.78	0.75	0.075
		Group 3 (SW26)	0.77	0.74	4.37	0.283
	Validation	Group 1 (SW32)	0.79	0.78	1.17	0.529
		Group 2 (SW33)	0.75	0.70	0.41	0.192
		Group 3 (SW26)	0.78	0.77	0.77	0.036
Pseudo ungauged watershed		Group 1 (SW25)	0.80	0.74	0.33	0.005
		Group 2 (SW42)	0.84	0.82	1.30	0.038
		Group 3 (SW29)	0.86	0.81	1.27	0.256

We then used the calibrated parameters in the validation of the same donor watersheds for the 2012–2014 time period. The obtained hydrographs are shown in Figs. 6, 7, and 8; and the calculated statistical coefficient is indicated in Fig. 9 and Table 5. Results showed that the model performance in the Gilan Watershed (SW33) was higher than in other watersheds, probably because of the comparatively large watershed area. The SWAT model generally performs best in large watersheds^55,56^.

### Streamflow prediction in the ungauged watersheds

**Figure 10:**
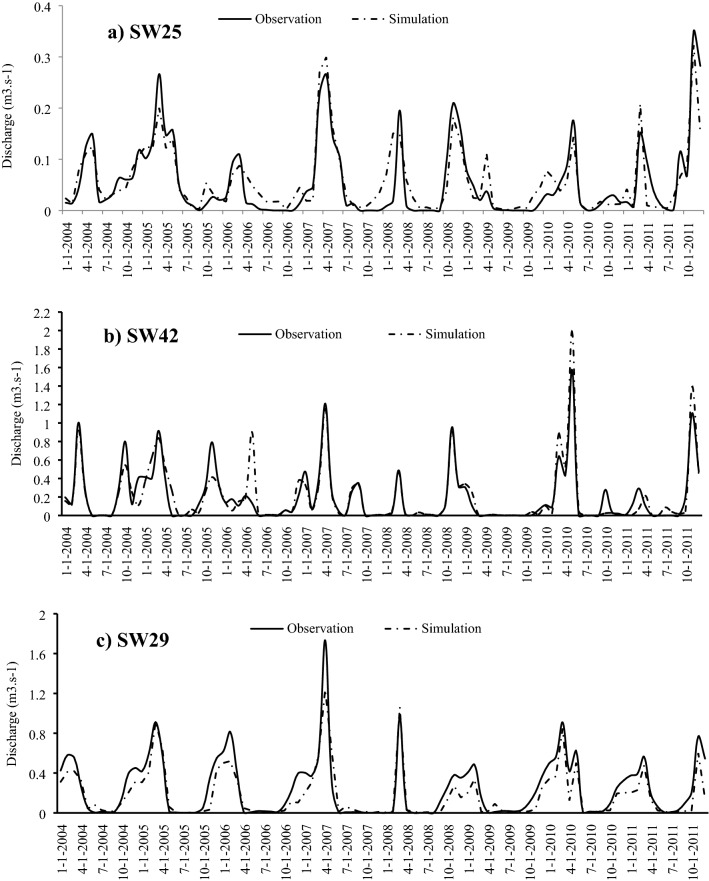
Streamflow simulation in the pseudo ungauged watersheds: (**a**) SW25, (**b**) SW42, (**c**) SW29.

After calibration and validation of the SWAT in gauged (donor) watersheds, it was run for the three pseudo watersheds in the three groups (SW25, SW42, and SW29). The calculated statistical coefficients indicated that the SWAT model had a very good performance in estimating the streamflow in pseudo ungauged watersheds (Table 5; Fig. 10). The good performance on the pseudo ungauged watersheds indicates the usefulness of the model for estimating the streamflow in ungauged watersheds (which are in the same group) (Table 5). The model is somewhat poor at simulating peak flows in April month. This issue is mainly because of the effect of snow melting in this month that leads to increasing streamflow. The observed streamflow in this month is higher than the simulated value. This issue mainly is mentioned in the watersheds covered by snow such as Vilaysane et al.^33^ (in the Xedone River Basin, Thai) and Choubin et al.^12^ (in the Karkheh River Basin, Iran). The time and the value of most of the peak flow events were simulated with high precision that shows the used SCS equation in the SWAT model has a high ability for simulation streamflow^12,33,36^. The overall results indicated a good performance of the SWAT model for regionalization objectives in ungauged watersheds. The obtained results of the model simulation in ungauged watersheds could have many implications on water resource management such as having an estimation of the streamflow average volume, peak flow time, peak flow volume, and etc.^52,57^. These data are also very important in determining the type and location of watershed management operation.

Therefore, we ran the model in the ungauged watersheds SW43, SW30, and SW3, in groups 1, 2, and 3, respectively. Ungauged watershed SW43 in group 1, which has a 15.5 km^2^ area and an elevation range between 1550 and 2200 m, is considered a mountain watershed. The average simulated streamflow in this ungauged watershed is 0.07 m^3^ s^−1^, and the minimum and maximum streamflow are 0.01 and 0.2 m^3^ s^−1^. In the second group, the ungauged watershed SW30, having a 127.6 km^2^ area and elevation between 1575 and 3140 m, the average simulated streamflow is 0.16 m^3^ s^−1^; and the range of streamflow is between 0.04 and 0.4 m^3^ s^−1^. The SW3 has an area of 78.4 km^2^ and was selected as an ungauged watershed in the third group. The elevation in this watershed is between 1123 and 1734 m. The average of simulated streamflow in this watershed is 0.1 m^3^ s^−1^, which is more than SW43 streamflow and less than SW30. The minimum and maximum streamflow values in this watershed are 0 and 0.84 m^3^ s^−1^. Considering the characteristics of these watersheds, the simulated stream flows are acceptable.

## Conclusions

We found that the SWAT model was useful in developing a regionalization method to simulate streamflow in ungauged watersheds in the Gharehsoo watershed in Iran. In this paper, we used fuzzy c-means clustering to partition the 46 watersheds into three homogenous groups that were similar to seven hydro-geomorphic parameters: area, maximum elevation, minimum elevation, slope length, slope, rainfall, and temperature. The model was calibrated and validated using optimal parameters and data from one gauged donor watershed in each group, then tested on another gauged watershed from the same group (considered as a pseudo ungauged watershed). Values of R^2^ and NSE > 0.7 indicated that the model performance in estimating streamflow in all three groups was acceptable for applying to the ungauged watersheds in the region. In most of the arid and semi-arid regions, the small watersheds are ungauged therefore this approach provides a reliable method for streamflow estimation. Short hydrologic data availability was the main limitation of this study. Experience of the model with different dry and wet periods in the calibration period can affect the model performance. Separating the dry and wet periods or considering a longer period may improve the model performance, which is recommended for future studies. The impact of water resources in developing agriculture products in arid and semi-arid regions is vital and, in these regions, accurate streamflow simulation is important for the management of the watersheds.
